# Clinical prediction models for diagnosis of COVID-19 among adult patients: a validation and agreement study

**DOI:** 10.1186/s12879-022-07420-4

**Published:** 2022-05-14

**Authors:** Nadia Dardenne, Médéa Locquet, Anh Nguyet Diep, Allison Gilbert, Sophie Delrez, Charlotte Beaudart, Christian Brabant, Alexandre Ghuysen, Anne-Françoise Donneau, Olivier Bruyère

**Affiliations:** 1 Biostatistics Unit, University of Liège, Quartier Hôpital, Av. Hippocrate 13, CHU B23, 4000 Liège, Belgium; 2 WHO Collaborating Centre for Public Health, Aspects of Musculo-Skeletal Health and Ageing, Research Unit in Public Health, Epidemiology and Health, Economics, University of Liège, Quartier Hôpital, Av. Hippocrate 13, CHU B23, 4000 Liège, Belgium; 3 Emergency Department, University Hospital Center, Avenue de L’Hôpital 1, 4000 Liège, Belgium

**Keywords:** COVID-19, Patients’ triage, Prediction models, Validation study, Agreement

## Abstract

**Background:**

Since the beginning of the pandemic, hospitals have been constantly overcrowded, with several observed waves of infected cases and hospitalisations. To avoid as much as possible this situation, efficient tools to facilitate the diagnosis of COVID-19 are needed.

**Objective:**

To evaluate and compare prediction models to diagnose COVID-19 identified in a systematic review published recently using performance indicators such as discrimination and calibration measures.

**Methods:**

A total of 1618 adult patients present at two Emergency Department triage centers and for whom qRT-PCR tests had been performed were included in this study. Six previously published models were reconstructed and assessed using diagnostic tests as sensitivity (Se) and negative predictive value (NPV), discrimination (Area Under the Roc Curve (AUROC)) and calibration measures. Agreement was also measured between them using Kappa’s coefficient and IntraClass Correlation Coefficient (ICC). A sensitivity analysis has been conducted by waves of patients.

**Results:**

Among the 6 selected models, those based only on symptoms and/or risk exposure were found to be less efficient than those based on biological parameters and/or radiological examination with smallest AUROC values (< 0.80). However, all models showed good calibration and values above > 0.75 for Se and NPV but poor agreement (Kappa and ICC < 0.5) between them. The results of the first wave were similar to those of the second wave.

**Conclusion:**

Although quite acceptable and similar results were found between all models, the importance of radiological examination was also emphasized, making it difficult to find an appropriate triage system to classify patients at risk for COVID-19.

**Supplementary Information:**

The online version contains supplementary material available at 10.1186/s12879-022-07420-4.

## Background

Coronavirus disease 2019 (COVID-19) is an infectious disease caused by severe acute respiratory syndrome coronavirus 2 (SARS-CoV-2). The first cases of COVID-19 have been identified in December 2019 in Wuhan, China and the World Health Organization (WHO) declared COVID-19 a global pandemic on March 11, 2020. To date, more than 180 million cases have been confirmed and more than 4 millions of deaths are to be deplored [1]. Symptoms range from mild (nothing for asymptomatic patients, fever, cough) to severe (shortness of breath or difficulty in breathing) and can lead to hospitalisation and admission to intensive care units (ICUs) or even death [2]. The need to increase capacity and reorganise health care departments has become rapidly apparent as the number of potentially infected patients is on the increase. Since the beginning of the pandemic, hospitals have been continuously overcrowded, with several observed waves of infected cases and hospitalisations. For example, in Belgium, nearly 7500 beds were occupied in hospitals, including more than 1400 in intensive care, for a maximum of 2800 beds, a capacity that was increased during this period, which is a record during the second wave in November 2020 [3]. To avoid at maximum this overcrowding and to take care of the patients in the best possible way, Emergency Departments (EDs) need to have efficient tools to confirm the diagnosis of COVID-19.

Until now, the most common first-line screening test used as gold standard to diagnose case of COVID-19 remains the quantitative real-time reverse transcriptase polymerase chain reaction (qRT-PCR) even if other screening tools based, for instance, on saliva or deep throat sputum (DTS) seemed to show similar efficacy [4]. This test has the advantage to be realised quickly with proving high sensitivity, but with observed false-negative results [5, 6], and can produce results in 3–4 h [7]. Unfortunately, in practice and due to a high number of tests realised, the waiting time for the results is generally longer, up to more than 24 h in hospitals. As suggested by Soedarsono et al. [5] to prevent the false-negative results, a combination of qRT-PCR with clinical, radiological or serological examinations could further support the clinicians in the triage of patients at high risk of COVID-19.

Systematic reviews or validation of prediction and prognostic models have been already realised in other studies [8, 9]. In Wynants et al. [8], the authors presented 33 diagnostic models for predicting COVID-19 with discrimination and calibration measures, but, although suggested but not realised by the authors themselves, these models have not been validated in other data sets or by independent investigators. In a recent external validation by Gupta et al. [9], the authors focused on patients confirmed with COVID-19 and different outcomes including mortality, ICU admission or progression to severe COVID-19. Their primary objective was to detect patient at high risk of deterioration, not to focus on an efficient tool to classify patients at high risk of COVID-19. In both studies, patients from only one specific wave were enrolled.

Based on a new systematic review of recent literature, the objectives of our research are to evaluate and compare prediction models for diagnosis of COVID-19 using an independent dataset including several waves of the pandemic. Results from our systematic review are already available and published in Archives of Public Health and entitled “A systematic review of prediction models to diagnose COVID-19 in adults admitted to healthcare centers” [10]. This paper presents the selected models that will be validated in the present study. The evaluation of these models will be based on criteria of performance indicators as discrimination and calibration measures. Several agreement indexes will be also computed for purposes of model comparison. Moreover, as mentioned above, the employed database incorporated a lot of information over two complete waves (from March to June 2020 and from September 2020 to January 2021) of COVID-19 infected cases in Belgium and allowed the construction of alternative models to perform sensitivity analyses. In so doing, the results achieved are to provide an overview of the latest models as a basis for decision making, to guide and advance further studies in COVID-19 model development, and to confirm and/or verify the conclusions from Wynants et al. [8] that all COVID-19 diagnostic models to date are poorly developed or useless. Finally, some easy-to-use models could be highlighted to help clinicians classify patients at high risk of COVID-19.

## Methods

### Presentation of the selected models of the systematic review

Thirteen articles were included in our systematic review “A systematic review of prediction models to diagnose COVID-19 in adults admitted to healthcare centers” [10] and all were performed in 2020. Each study proposed diagnostic models for COVID-19 based on socio-demographics, clinical symptoms, blood tests, or other characteristics that were compared to the qRT-PCR test. The number of variables included in the model varied from 4 to 15. The presence of fever appeared in 7 models, the blood value of eosinophils in 6 models, and C-reactive protein (CRP) in 5 models. Four studies included comorbidities, gender (male) or chest X-ray as a predictor in their models. Finally, age, cough, white blood cells (WBC) were significant predictors in three out of 13 studies and lymphocytes was present in two out of the 13 studies. It can be noted that some variables can be directly collected while others require more time for their investigation. Sample sizes varied from 100 to 172 754 subjects and most studies were conducted at a single site or institution. Most of the models were developed using logistic regressions. From these logistic regressions, some authors developed a score and derived cut-off values. Models such as XGBoost, random forest and machine learning were also applied. All presented classification measures, with a wide range of sensitivity and specificity values depending on the model and 12 presented a discrimination measure. All models performed well to identify patients at risk of COVID-19 but only one proceeds to an external validation. The risk of bias was estimated as low for all models using the PROBAST tool [11].

Among these 13 articles, six were kept in this study to calculate scores, cut-off values and fit models. The other articles were discarded due to missing information > 20% and/or the impossibility to calculate the score or to fit the model due to the methodology used and/or lack of information despite contacts with the authors as it will be explained in detail in the following sections. As mentioned in [10], it can also be noted that the collected variables were sometimes country-specific and cannot be obtained if the model is to be put into use in a setting other than the research context. They are studies from Vieceli et al. [12], Tordjman et al. [13], Kurstjens et al. [14], Aldobyany et al. [15], Nakakubo et al. [16] and Fink et al. [17] and are presented in detail in the Additional file 1: Appendix A1. For most of them, a score and cut-off values could be obtained but a binary logistic regression was only available for three studies [12, 13, 17]. A score and cut-off value had to be refitted due to missing information and another missing variable was replaced by its median value to fit the logistic regression model. For the score derived from Nakakubo et al., the two categories “moderate and high risk” were combined due to few subjects in the last category in the sample.

### Study population

Data in the present study have been extracted from the Medical and Economic Information Service (SIME) of the University Hospital Center of Liège (CHU Liège) and included patients present at the two ED triage centers [18] of the CHU (Sart Tilman and Notre-Dame des Bruyères) with suspicion of COVID-19. Data were collected during the period from March 2, 2020, to January 31, 2021. The number of patients was 8033. This period primarily covered two complete waves of cases and patient admissions in Belgium [3]: from March 2020 to June 2020 (wave 1) and from September 2020 to January 2021 (wave 2).

Socio-demographic information (age and gender) as well as comorbidities (cardiac disease, immunosuppression, renal failure), symptoms (fever, dry or wet cough, dyspnea, diarrhea), blood parameters (lactic acid dehydrogenase LDH, CRP, procalcitonin, lymphocytes or lymphocytes count ALC, basophils, ferritin, leukocytes, neutrophils or neutrophils count ANC), radiology exams, particularly chest X-ray results, were collected in the database. Socio-demographic information and clinical symptoms were factors easily available at ED’s admission whereas hospital diagnostic resources required a more important time-to-results. In addition, radiological resources were not recommended to all patients, as their clinical presentation could not require this type of work-up. The outcome was confirmed or unconfirmed COVID-19 case using a qRT-PCR. Two different qRT-PCR tests were used during these periods: one adapted from the protocol described by Corman et al. [19]; and a second was a commercial assay using the cobas^®^ 6800 platform (Roche) [18]. Patients for whom no qRT-PCR test was realised, aged < 18 years and for whom no biological parameters were not included in the analysis, representing 80% of the original dataset.

Eventually, 1618 patients (20% from the original database) were included in this study, with no pregnant women and with 32.1% positive cases to the qRT-PCR.

### Statistical analysis

Results were expressed as numbers and frequencies for qualitative parameters and as mean and standard deviation (SD), median (P50) and interquartile range (IQR, P25-P75) and range (Min–Max) for quantitative parameters, globally and by groups, namely positive and negative confirmed COVID-19 patients. The normality of the distribution of the quantitative parameters was investigated using the mean-median comparison, the histogram and Quantile–Quantile plot and tested with the Shapiro–Wilk hypothesis test.

For all models and scores, discrimination was assessed by the Area Under the Receiver Operating characteristic Curve (AUROC). Values could range from 0 to 1 where AUROC of 0.5 suggests no discrimination, values from 0.7 to 0.8 are considered acceptable, from 0.8 to 0.9 as excellent, and more than 0.9 as outstanding [20]. For models that provided a cut-off value, sensitivity (Se), specificity (Sp), positive and negative predictive values (PPV and NPV respectively) were also calculated with 95% confidence interval (95CI%).

For models where information was available to calculate outcome probabilities, model calibration was assessed by means of the Brier score, values can range from 0 for a perfect model to 0.25 for a non-informative model [21, 22] and represents a measure of accuracy, and by calibration of predicted probabilities versus observed probabilities using LOESS- smoothed plot. Results were reported with calibration slopes and intercept (calibration-in-the-large). A perfect calibration slope is equal to 1 while slopes < 1 indicate an underestimation of low risk and overestimation of high risk and slopes > 1 means underestimation of high risk and overestimation of low risk. The estimated regression intercept represents the overall miscalibration, where 0 indicates good calibration, > 0 denotes an average underestimation, and < 0 denotes an average overestimation [23]. For models where information for intercept was missing, we calculated the intercept using the model linear predictors as an offset term as suggested by Gupta et al. [9].

A sensitivity analysis was also conducted to compare Se, Sp, PPV, NPV but also discrimination and calibration measures for each selected model when using the complete data set and data set where patients were excluded from (1) wave 1 and between the two waves and (2) wave 2 and between the two waves.

Agreement between models was tested by means absolute and relative measures. For continuous scores, a pairwise comparison using Bland–Altman (BA) with limits of agreement (LOA) and two-way fixed IntraClass Correlation Coefficient (ICC (A, 1) [24]) with 95CI%. Values less than 0.5 are indicative of poor reliability, values between 0.5 and 0.75 indicate moderate reliability, values between 0.75 and 0.9 indicate good reliability, and values greater than 0.90 indicate excellent reliability [25]. As scores had different value range, they were rescaled (mean/standard deviation) for these calculations. For binary or categorical score, Cohen’s Kappa was computed [26]. Values > 0.6 indicates substantial agreement [27].

If a maximum 20% of the information to calculate a score or to fit a model was unobtainable from the data, calculation was based on the available variables [28]. Scores and possible cut-off values were refitted to the actual number of variables. To fit models, missing variables were replaced by the mean, or the median value given in the original article. Where more than 20% of variables were missing, the score/model was discarded from this study.

The amount of missing data varied from 0.2% to 63%. Multiple imputation using the Fully Conditional Specification (FCS) method [29] was applied and all statistical analyses, diagnostic values, discrimination, calibration and agreement, were realised on the 60 generated data sets. The Rubin’s rules [30] was applied to pool the obtained results.

Results were significant at the 5% critical level (p < 0.05). The statistical analyses were carried out using SAS (version 9.4 for Windows) statistical package and R (version 4.0) with particular packages rms [31], CalibrationCurve [32], BlandAltmanLeh [33], and multiagree [34] and more common iir [35] and psych [36].

## Results

### Description of the data

All variables used in this study are presented in Table 1 and correspond to those included in the 6 selected models. Among the 1618 patients presenting to the two ED triage centers and enrolled in this study, 54.6% were men with a median age of 73 (IQR: 62–82) years, 80.1% had an abnormal radiology and 32.1% were positive to the qRT-PCR test. Information about comorbidities, symptoms and biological parameters are also provided, for all patients and by results of qRT-PCR test. Highest percentages of missing values appeared in the information on comorbidities and symptoms. For some scores/models, transformation of biological parameters was made due to differences in measure units from the in original studies. Presence of fever, dry cough and abnormal result of chest X-ray were more present in positive patients. For biological parameters, median values of LDH, CRP and ferritin were higher in positive patients while median values of leukocytes and neutrophils were lower.

**Table 1:** Descriptive analysis for all parameters used in scores/formulas, globally and by results from qRT-PCR screening test

		qRT-PCR test			
		All	Positive	Negative	% missing
Demographics					
Age (years)	Total	1618	519	1099	0%
	P50 (P25–P75)	73.00 (62–82)	72.00 (62–81)	73.00 (62–82)	
Gender	Total	1618	519	1099	0%
	Male (n, %)	883 (54.6)	320 (61.7)	563 (51.2)	
	Female (n, %)	735 (45.4)	199 (38.3)	536 (48.8)	
Period	Total	1618	519	1099	0%
	Wave 1 (n, %)	588 (36.3)	146 (28.1)	442 (40.2)	
	Between (n, %)	204 (12.6)	15 (2.9)	189 (17.2)	
	Wave 2 (n, %)	826 (51.1)	358 (69.0)	468 (42.6)	
Comorbidities					
Cardiac disease	Total	593	268	325	63.3%
	Yes (n, %)	200 (33.7)	72 (26.9)	128 (39.4)	
Immunosuppression	Total	593	268	325	63.3%
	Yes (n, %)	38 (6.4)	11 (4.1)	27 (8.3)	
Renal failure	Total	594	268	326	63.3%
	Yes (n, %)	48 (8.1)	17 (6.3)	31 (9.5)	
Symptoms					
Fever	Total	608	272	336	62.4%
	Yes (n, %)	282 (46.4)	169 (62.1)	113 (33.6)	
Dry cough	Total	607	272	335	62.4%
	Yes (n, %)	220 (36.2)	125 (46.0)	95 (28.4)	
Wet cough	Total	607	272	335	
	Yes (n, %)	106 (17.5)	45 (16.5)	61 (18.2)	
Dyspnea	Total	608	272	336	62.4%
	Yes (n, %)	403 (66.3)	192 (70.6)	211 (62.8)	
Diarrhea	Total	607	272	335	62.4%
	Yes (n, %)	128 (21.1)	69 (25.4)	59 (17.6)	
Biological parameters					
LDH U/L	Total	1618	519	1099	0%
	P50 (P25–P75)	275 (219–368)	349 (267–457)	252 (205–318)	
CRP mg/L	Total	1618	519	1099	0%
	P50 (P25–P75)	60.40 (15.1–140.9)	86.40 (43.6–164.2)	41.40 (9.50–129.9)	
Procalcitonin µg/L	Total	1615	588	202	0.2%
	P50 (P25–P75)	0.10 (0.04–0.34)	0.13 (0.06–0.34)	0.09 (0.04–0.35)	
Lymphocytes 10^3^/mm^3^	Total	1618	519	1099	0%
	P50 (P25–P75)	1.01 (0.66–1.57)	0.85 (0.61–1.19)	1.12 (0.71–1.74)	
Basophils 10^3^/mm^3^	Total	1618	519	1099	0%
	P50 (P25–P75)	0.03 (0.01–0.05)	0.02 (0.01–0.03)	0.03 (0.02–0.05)	
Eosinophils 10^3^/mm^3^	Total	1618	519	1099	0%
	P50 (P25–P75)	0.03 (0.01–0.12)	0.01 (0.00–0.03)	0.07 (0.01–0.16)	
Ferritin µg/L	Total	1614	519	1099	0.2%
	P50 (P25–P75)	332.5 (145.1–762.7)	702.00 (322.4–1441.4)	248.28 (108.8–530)	
Leukocytes 10^3^/mm^3^	Total	1618	519	1099	0%
	P50 (P25–P75)	9.40 (6.61–12.76)	7.02 (5.00–10.26)	10.40 (7.83–13.90)	
Neutrophils 10^3^/mm^3^	Total	1618	519	1099	0%
	P50 (P25–P75)	7.09 (4.68–10.28)	5.46 (3.50–8.51)	8.03 (5.50–11.48)	
Chest X-ray					
Radiological anomaly	Total	1475	492	983	8.8%
	No (n, %)	294 (19.9)	37 (7.5)	257 (26.1)	
	Yes (n, %)	1181 (80.1)	455 (92.5)	726 (73.9)	
Finding (n, %)	Total	1475	492	983	8.8%
	No atypical signs	825 (55.9)	106 (21.5)	719 (73.1)	
	Subpleural or lower lung dominant distribution	104 (7.1)	56 (11.4)	48 (4.9)	
	Multilobar or bilateral lesion	458 (31.1)	301 (61.2)	157 (16.0)	
	GGO with or without consolidation	88 (6.0)	29 (5.9)	59 (6.0)	
Alternative diagnosis (n, %)	Total	1618	519	1099	0%
	More likely other diagnosis	309 (19.1)	15 (2.9)	294 (26.8)	
	Hard to determine	1055 (65.2)	379 (73.0)	676 (61.5)	
	More likely COVID-19	254 (15.7)	125 (24.1)	129 (11.7)	
Other finding (n, %)	Total	1475	492	983	8.8%
	No infiltrate	861 (58.4)	122 (24.8)	739 (75.2)	
	Unilateral infiltrate	129 (8.7)	48 (9.8)	81 (8.2)	
	Bilateral infiltrate	485 (32.9)	322 (65.4)	163 (16.6)	

### Diagnostic tests results

By applying the six selected models on the employed dataset, the results revealed that the smallest value for Se was given by the cut-off value derived from the score from Nakakubo et al. [16] but, globally, all studies provided, according to the choice of the cut-off value, quite similar results. For studies providing only one cut-off value, i.e. Vieceli et al. [12] and Nakakubo et al. [16], the values for Se and NPV were higher (> 0.7) than those for Sp and PPV (< 0.7). When several cut-offs values were proposed, namely in Aldobyany et al. [15], Tordjman et al. [13] and Kurstjens et al. [14], Se and PPV were higher for smaller values of cut-off while Sp and NPV were higher for greater values. Cut-offs values from 1 to 3 in Tordjam et al. [13] gave results > 0.8 for Se and NPV. Same remarks could be made for cut-off values from 2 to 5 in Kurtsjens et al. [14]. Finally, multiple imputation gave robust results when compared with those from the whole sample (Table 2).

**Table 2:** Sensitivity, specificity, positive and negative predictive values for scores from selected articles with given cut-offs

		qRT-PCR test									
		All		Positive		Negative		Diagnostic test results (Rubin’s rules)			
		Total	n (%)	Total	n (%)	Total	n (%)	Se (CI95%)	Sp (95CI%)	PPV (95CI%)	NPV (95CI%)
Vieceli et al.		1475		492		983					
Cut-off value	≥ 5		847 (57.4)		418 (85.0)		429 (43.6)	0.84 (0.80–0.87)	0.57 (0.54–0.60)	0.48 (0.45–0.51)	0.88 (0.86–0.91)
Tordjman et al.		1618		519		1099					
Cut-off value	≥ 0		1618 (100.0)		519 (100.0)		1099 (100.0)	/	/	/	/
	≥ 1		1427 (88.2)		503 (96.9)		924 (84.1)	0.97 (0.95–0.98)	0.16 (0.14–0.18)	0.35 (0.33–0.38)	0.92 (0.87–0.95)
	≥ 2		1224 (75.6)		475 (91.5)		749 (68.2)	0.92 (0.89–0.94)	0.32 (0.29–0.35)	0.39 (0.36–0.42)	0.89 (0.85–0.92)
	≥ 3		952 (58.8)		419 (80.7)		533 (48.5)	0.81 (0.77–0.84)	0.52 (0.49–0.54)	0.44 (0.41–0.47)	0.85 (0.82–0.88)
	≥ 4		649 (40.1)		330 (63.6)		319 (29.0)	0.64 (0.59–0.68)	0.71 (0.68–0.74)	0.51 (0.47–0.55)	0.80 (0.78–0.83)
	≥ 5		204 (12.6)		145 (27.9)		59 (5.4)	0.28 (0.24–0.32)	0.95 (0.93–0.96)	0.71 (0.64–0.77)	0.74 (0.71–0.76)
Kurstjens et al.		1472		489		983					
Cut-off value	> 2		1123 (76.3)		468 (95.7)		655 (66.6)	0.96 (0.94–0.98)	0.33 (0.30–0.36)	0.40 (0.38–0.43)	0.94 (0.92–0.97)
	> 3		1010 (68.6)		457 (93.5)		553 (56.3)	0.93 (0.91–0.95)	0.44 (0.41–0.47)	0.44 (0.41–0.47)	0.93 (0.91–0.95)
	> 4		884 (60.1)		443 (90.6)		441 (44.9)	0.90 (0.87–0.93)	0.55 (0.52–0.58)	0.49 (0.45–0.52)	0.92 (0.90–0.94)
	≥ 5		756 (51.4)		423 (86.5)		333 (33.9)	0.86 (0.83–0.89)	0.66 (0.63–0.69)	0.54 (0.51–0.58)	0.91 (0.89–0.93)
	≥ 9		440 (29.9)		333 (68.1)		107 (10.9)	0.66 (0.62–0.71)	0.89 (0.87–0.91)	0.74 (0.70–0.85)	0.85 (0.83–0.87)
	≥ 10		355 (24.1)		281 (57.5)		74 (7.5)	0.56 (0.52–0.60)	0.92 (0.91–0.94)	0.78 (0.74–0.82)	0.82 (0.80–0.84)
	≥ 11		275 (18.7)		229 (46.8)		46 (4.7)	0.45 (0.41–0.50)	0.95 (0.94–0.96)	0.82 (0.78–0.87)	0.79 (0.76–0.81)
	≥ 12		200 (13.6)		176 (36.0)		24 (2.4)	0.35 (0.30–0.39)	0.98 (0.97–0.99)	0.87 (0.82–0.92)	0.76 (0.74–0.78)
Aldobyany et al.		502		224		278					
Cut-off value	≥ 3		384 (76.5)		188 (83.9)		196 (70.5)	0.81 (0.77–0.84)	0.28 (0.25–0.30)	0.35 (0.32–0.37)	0.75 (0.71–0.80)
	≥ 4		300 (59.8)		168 (75.0)		132 (47.5)	0.69 (0.65–0.73)	0.47 (0.44–0.50)	0.38 (0.35–0.41)	0.76 (0.73–0.80)
Nakakubo et al.		1615		519		1096					
Cut-off value	> 5		762 (47.2)		397 (76.5)		365 (33.3)	0.78 (0.74–0.82)	0.64 (0.61–0.67)	0.51 (0.47–0.54)	0.86 (0.84–0.89)

### Discrimination and calibration measures

**Fig. 1:**
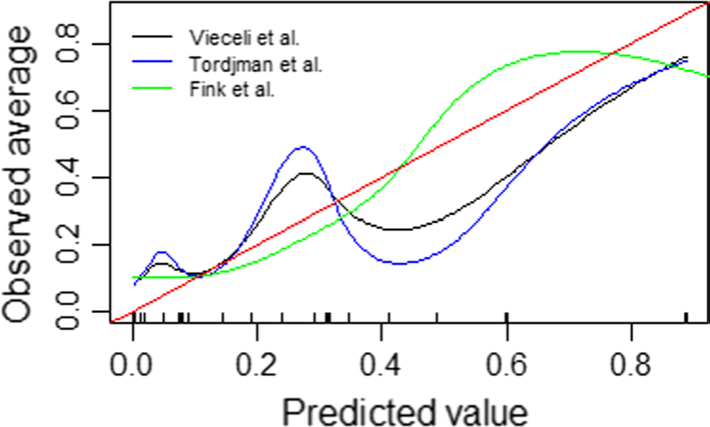
Calibration plots for all models

Discrimination and calibrations results are given in Table 3. Values of AUROC for all scores or models were acceptable (> 0.7) or excellent (> 0.8) excepted for the score derived from Aldobyany et al. [15] where the AUROC was equal to 0.60. Brier score were similar (0.18–0.19) and showed poor accuracy. All models showed however good calibration with calibration intercept equal to 0. Models derived from Vieceli et al. [12] and Tordjam et al. [13] tended to overestimated high risk (calibration slope < 1) while model derived from Fink et al. [17] tented to underestimate it (calibration slope > 1). Calibration plots were depicted in Fig. 1 and confirmed these findings. Values of AUROC seemed to be robust after multiple imputation according to the median and IQR values of scores in each group, positive and negative cases, in the sample.

**Table 3:** AUROC and calibration measures for scores and formulas from selected articles with cut-offs

	qRT-PCR test									
	All		Positive		Negative		Discrimination and calibration results (Rubin’s rules)			
									Calibration	
	N	P50 (P25–P75)	N	P50 (P25–P75)	N	P50 (P25–P75)	AUROC (95CI%)	Brier score	Slope (95 CI%)	Intercept (95 CI%)
Vieceli et al	1475		492		983					
Score (0–9)		6 (4–7)		7 (6–9)		4 (4–6)	0.77 (0.74–0.79)			
Model							0.77 (0.74–0.89)	0.18	0.55 (0.48–0.62)	− 0.00 (− 0.14 to 0.14)
Original value							*0.85 (0.77–0.92)*			
Tordjman et al	1618		519		1099					
Score (0–5)		3 (2–4)		4 (3–5)		2 (1–4)	0.73 (0.71–0.76)			
Model							0.74 (0.72–0.77)	0.19	0.40 (0.35–0.46)	− 0.0 (− 0.14 to 0.14)
Original value							*0.92 (0.89–0.95)*			
Kurstjens et al	1472		489		983					
Score (0–14)		6 (3–14)		10 (7–12)		4 (2–6)	0.84 (0.83–0.87)			
Original value							*0.94 (0.91–0.96)*			
Aldobyany et al	502		224		278					
Score (0–8)		4 (3–5)		5 (3.5–6)		3 (2–5)	0.60 (0.57–0.63)			
Original value							*0.60 (0.57–0.64)*			
Nakakubo et al	1615		519		1096					
Score (0–11)		4 (3–6)		6 (5–6)		4 (3–5)	0.78 (0.76–0.81)			
Original value							*/*			
Fink et al	564		259		305		0.78 (0.75–0.80)	0.18	1.13 (0.99–1.27)	0.00 (− 0.11 to 0.11)
Model										
Original value							*0.85 (0.81–0.90)*

### Agreement measures

**Fig. 2:**
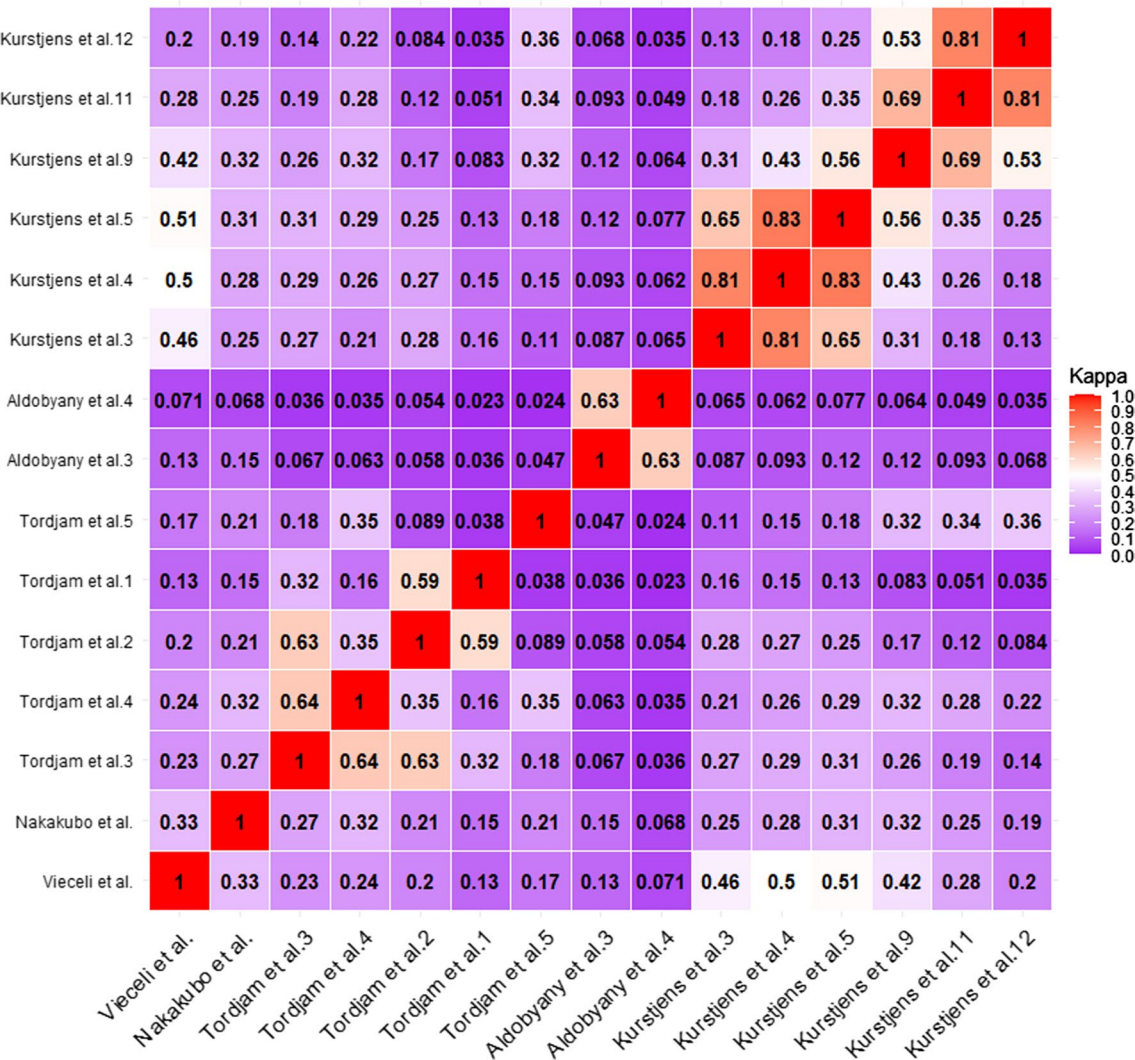
Cohen’s Kappa between cut-off scores (name of the first author of the article followed by the cut-off value)

Agreement between scores (rescaled) and binary were also assessed. Results are presented in Table 4 for ICC and Bland–Altman and in a heatmap (Fig. 2) for the Kappa. Except between the scores and cut-off scores derived from Vieceli et al. [37] and Kurstjens et al. [14] where ICC and Kappa were higher or equal to 0.5, the other scores and cut-off scores showed poor agreement. Indeed, ICC varied from to 0.16 to 0.44, Kappa from 0.02 to 0.46. Higher values of Kappa (> 0.6) were only observed between the cut-off score derived from the same original score. Even if the mean differences were close to 0, the limits of agreement (LOA) were very large according to the fact that score were rescaled in order to be compared.

**Table 4:** Agreement between score (rescaled mean/standard deviation)—ICC and BA (Rubin’s rule)

	Vieceli et al.	Nakakubo et al.	Tordjman et al.	Aldobyany et al.	Kurstjen et al
Vieceli et al.		*ICC I95CI%): 0.43 (0.38–0.47)*	*ICC (95CI%): 0.37 (0.32–0.41)*	*ICC (95CI%): 0.17 (0.13–0.22)*	*ICC (95CI%): 0.61 (0.58–0.65)*
Nakakubo et al.	MD ± LOA: 0.00 ± 2.10		*ICC (95CI%): 0.38 (0.35–0.43)*	*ICC (95CI%): 0.16 (0.12–0.21)*	*ICC (95CI%): 0.44 (0.40–0.48)*
Tordjman et al.	MD ± LOA: 0.00 ± 2.20	MD ± LOA: 0.00 ± 2.17		*ICC (95CI%): 0.10 (0.055–0.15)*	*ICC (95CI%): 0.46 (0.43–0.50)*
Aldobyany et al.	MD ± LOA: 0.00 ± 2.52	MD ± LOA: 0.00 ± 2.53	MD ± LOA: 0.00 ± 2.62		*ICC (95CI%): 0.17 (0.12–0.22)*
Kurstjen et al.	MD ± LOA: 0.00 ± 1.72	MD ± LOA: 0.00 ± 2.08	MD ± LOA: 0.00 ± 2.03	MD ± LOA: 0.00 ± 2.53	

ICC (95CI%): IntraClass Coefficient (Confidence interval 95%)

MD ± LOA: mean difference ± limit of agreement

### Sensitivity analysis

Finally, to conduct a sensitivity analysis and to check if results remained stable according to different groups of patients, diagnostic tests results, discrimination and calibration measures were also calculated for each wave of patients. Results are given in the Additional file 1: Appendix A2 for Se, SP, PPV and NPV and in Additional file 1: Table A3 for discrimination and calibration measures. All calculations remained stable over time. Moreover, the same observations as those made when all the patients were taken into account in the analyses were still valid.

## Discussion

### The performance of the studied scores and models

All AUROC values obtained in this study were close but generally smaller to those mentioned in the original paper. For example, in Fink et al. [17], original AUROC value was equal to 0.85 (0.81–0.90) which was a better result than in this study (0.78 (0.75–0.80)). It could be explained by the missing information about one variable (highest FiO_2_) to reconstruct the logistic regression model and that had to be replaced by the median value mentioned in the original article. Despite another missing information to calculate score derived from Aldobyany et al. [15], the obtained AUROC value in this study was similar. The missing information concerned the exposure risk and counted for 3 points out of 11. Globally, results remained acceptable but were excellent or outstanding in the original articles. For the binary regression models, all showed a good calibration with calibration intercept equal to 0 but two models tended to overestimate high risk while one tended to underestimate it. The Brier scores showed poor accuracy but they did not always perform well to evaluate clinical utility of diagnostic performance of these prediction models as mentioned in [40]. When comparing diagnostic tests results for these two studies, again, quite similar but smaller values for Se/NPV and/or Sp/PPV were found when compared to the results of the original articles. A cut-off value between 1 and 3 could be suggested to maximize the value of Se in [13] and, for the same reason, a cut-off of 3 in [15]. Unfortunately, no comparison could be made with results derived from Nakakubo et al. [16], because the original article proposed to classify patients in three groups (low, moderate and high risk). The last two groups were collapsed in this study and showed encouraging results for Se and NPV. All these results nuance or even contradict the critical remark of Wynants et al. [8] on the non-utility of this kind of model.

### The relative importance of different types of predictive variables

The variables used in the different scores are described in details in Additional file 1: Appendix A4. As mentioned above, except for one score, all models presented in this study showed acceptable (> 0.70) or excellent (> 0.80) values for AUROC. The score with the smallest value (0.60) was derived from Aldobyany et al. [15], the only article that did not use biological parameters or results from radiological examination, highlighting the importance of these parameters. Unfortunately, only a score was given, and no calibration measures could be calculated. The model derived from Tordjman et al. [13], which was also very simple with only four biological parameters and no radiological exam, showed an AUROC value equal to 0.74 and good and quite similar calibration results when compared to the other most sophisticated models where the result from chest X-ray was used. However, it tended to overestimate high risk. These findings showed that a clinical triage of patients based only on symptoms and/or risk exposure is less efficient than one based on biological parameters and/or radiological examination. This confirms the suggestion cited in [5] about the importance of a combination of qRT-PCR with clinical, radiological or serological in the triage of patients to prevent false-negative results. It also confirms the difficulty to build a diagnostic model that would be simple, effective and based on information immediately available upon hospital arrival or at the triage stage. If anamnestic data are easily and rapidly available, biological analysis are time consuming and even more radiological findings. A balance must be found between the accuracy and the time needed to calculate the prediction that should be faster that qRT-PCR method. The future solution could potentially come from innovative strategies combining point-of-care testing and artificial intelligence-driven models as described recently by Soltan et al. [41].

### Agreement between the scores and/or model

Finally, the scores, although calculated from some identical parameters and all showing acceptable results for diagnostic tests, discrimination and calibration, showed poor agreement between them. Indeed, ICC varied from to 0.16 to 0.44 while Kappa coefficients from 0.02 to 0.46. Moreover, the limit of agreement in the Bland–Altman analyses was very large. Poor agreement between the cut-off values could be explained by the fact that either the sensitivity values were very good at the expense of the specificity values or vice versa, and thus, the objectives, to maximize Se or Sp, were not the same for all cut-off scores. Another explanation could be the different predictor variables included in the scores and/or models, predictors with various clinical meaning. As mentioned by Gilbert et al. [42], the multiple predictive scores described currently in the literature present an important heterogeneity of the variables used (clinical, biological, radiological) related either to their recommended time-to-results, the availability of the data or resources in the concerned setting where the score was developed. In accordance, one important point that remains partially unanswered is the generalizability of these scores. It can be noted that the “best” value for agreement (ICC and Kappa > 0.5) was observed in two studies that have these two common parameters, LDH and results from chest X-ray, two parameters already recognised as important in the detection of COVID-19 [43].

### The robustness of the results

A sensitivity analysis by waves of patients, i.e. corresponding to the periods from March 2020 to June 2020 (wave 1) and from September 2020 to January 2021 (wave 2) [3], was performed. Results remained stable. However, in the original studies, all patients were recruited in the period corresponding to wave 1, depending on the country where the study was conducted. Even though the characteristics of the patients seemed to be different between the different periods (Additional file 1: Appendix Table A4), recent studies did not show any age or comorbidity differences between patients hospitalized during the first and second waves, although they pointed to a shorter hospitalization period in the second wave [44, 45]. The derived scores, cut-off scores and models could be considered robust according to the different periods of the pandemic.

### Strengths and limitations

This study evaluated and compared prediction models for diagnosis of COVID-19 identified through a systematic review of recent literature using performance indicators and agreement indexes, which, to the best of our knowledge, had not been done before. Moreover, the available database embodied a lot of information over two complete waves (from March to June 2020 and from September 2020 to January 2021) of cases in Belgium and allowed us to construct different models and to perform sensitivity analyses. Nevertheless, this study presents also certain important limitations. Indeed, from the original sample (8033), only 1618 (20%) patients were enrolled in the study due to the lack of information concerning the results from qRT-PCR test, biological parameters and radiological information. This information was necessary to calculate scores and/or fit models derived from the selected studies because (1) the result of qRT-PCR test was the outcome and (2) the other variables were predictors present in almost all the score and/or models selected in [10]. Despite this, the number of included patients met the sample size rules-of-thumb that suggests at least 100 events and 100 non-events [46, 47]. Secondly, roughly 60% of missing values were observed for comorbidities and symptoms. This is explained by the fact that, even if ED nurses and physicians were aware of the data collection, they were unfortunately overwhelmed by the number of patients. However, despite this amount of missing data, the results obtained in this study after multiple imputation seemed to be robust when compared to with those observed in the sample. To our knowledge, regarding robustness, similar observation was not mentioned in [9]. Another limitation was the inability to calculate score or to fit model for all articles from our previous systematic review. Indeed, more than 20% information like known exposure, Visual Analog Scale pain, Sequential Organ Failure Assessment or ethnicity required in [48–53] was not recorded in the database. Moreover, the modelling approach used in [54] could not be reproduced. So, only 6 articles out of 13 (46%) could be selected in this study, 5 presented score and one or more cut-off values, and only 3 presented results from binary regression models. Future external validation studies could address this issue of heterogeneity in model development and predictors by employing a more proactive and prospective approach in data collection. In so doing, more models could be externally validated with sufficient data, hence robust evidence being yielded. Finally, as already mentioned in [10], the choice of the studies included in the systemic review presents several biases like biases inherent in each selected studies but also specific to the systematic review like the database searched, the limitation of published with peer-reviewed studies and, of course, the period of time where this systematic review took place. Indeed, the COVID-19 pandemic has continued to evolve since then with new scientific and medical advances. That’s why, as suggested in [10], carrying out a living systematic review would be optimal.

## Conclusion

All derived scores, cut-off scores and models showed quite acceptable and similar results in terms of diagnostic tests, discrimination and calibration measures. Moreover, the values of the different measures calculated, although lower than those of the original articles, were still close and lead to similar conclusions. Despite this fact, poor agreement was found between the different derived scores and cut-off scores. Two scores had an advantage over others such that COVID-19 diagnosis could be calculated from rapid diagnostic as comorbidities or symptoms and/or blood sample. Nevertheless, these two models had the lowest but though acceptable values for discrimination and calibration measures, highlighting the importance of radiological examination to obtain more efficient models, which entails difficulties in specifying an easy-to-use tool to help clinicians to classify patients at risk of COVID-19.

## Supplementary Information

**Additional file 1**: Table A1. Presentation of the selected articles. **Table A2. **Sensitivity, specificity, predictive negative and positive value for scores from selected articles with cut-off values. **Table A3. **AUROC and calibration measures for scores and formulas from selected articles with cut-off values. **Table A4**. Descriptive analysis for all parameters used in scores/formulas, globally and by time period.

## Data Availability

Anonymized data are available upon reasonable request from the corresponding author.

## Abbreviations

95CI%: 95% Confidence interval
ALC: Lymphocytes count
ANC: Neutrophils count
AUROC: Area under the Roc curve
BA: Bland–Altman
CHU: University Hospital Center
COVID-19: Coronavirus disease 2019
CRP: C-reactive protein
ED: Emergency department
ICC: IntraClass coefficient
ICU: Intensive care unit
IQR: Interquartile range
LDH: Lactic acid dehydrogenase
LOA: Limits of agreement
Max: Maximum
MD: Mean difference
Min: Minimum
NPV: Negative predictive value
P25: Percentile 25
P50: Percentile 50, median
P75: Percentile 75
PPV: Negative predictive value
qRT-PCR: Quantitative real-time reverse transcriptase polymerase chain reaction
SARS-CoV-2: Severe acute respiratory syndrome coronavirus 2
SD: Standard deviation
Se: Sensitivity
SIME: Medical and Economic Information Service
Sp: Specificity
WBC: White blood cell
WHO: World Health Organization
