# Supplementary material for: Clinical prediction models for diagnosis of COVID-19 among adult patients: a validation and agreement study
Source: BMC Infect Dis. 2022 May 14;22:464. doi: 10.1186/s12879-022-07420-4 (PMC9107295; doi:10.1186/s12879-022-07420-4)
Supplement: Supplementary file 1 — Additional file 1: Table A1. Presentation of the selected articles. Table A2. Sensitivity, specificity, predictive negative and positive value for scores from selected articles with cut-off values. Table A3. AUROC and calibration measures for scores and formulas from selected articles with cut-off values. Table A4. Descriptive analysis for all parameters used in scores/formulas, globally and by time period. [file 12879_2022_7420_MOESM1_ESM.docx]

**Appendix**

| **Table A1. Presentation of the selected articles** | | | | |
| --- | --- | --- | --- | --- |
| Articles | Predictors | Modelling | Missing predictor | Available calculations |
| Vieceli et al [27] | Leukocytes  LDH  Any chest radiographic abnormality | Binary logistic regression | / | Logistic regression  Predictive score (0 –9)  Cut-off ≥ 5 |
|  |  |  |  |  |
| Tordjman et al [28] | Eosinophils  Lymphocytes  Neutrophils  Basophils | Binary logistic regression | / | Logistic regression  Predictive PARIS score (0 – 5)  Cut-off ≥ 0 to ≥ 5 |
|  |  |  |  |  |
| Kurstjens et al. (14) | Age  Sex  CRP  Ferritin  LDH  ALC  ANC  Chest X-ray | Python algorithm | / | Corona score (0 – 14)  Cut-off >2, >3, >4, ≥5, ≥9, ≥10, ≥11, ≥12 |
|  |  |  |  |  |
| Aldobyany et al [30] | Exposure risk  Fever  Cough  Dyspnea  Nausea, vomiting and or diarrhea  Chronis renal failure, CAD/heart failure, immunocompromised patient | Binary logistic regression | Exposure risk (3 points/11) | COVID - 19 triage score (0 – 11) refitted to (0 – 8)  Cut-off ≥ 4 and ≥ 5 refitted to ≥ 3 and ≥ 4 |
|  |  |  |  |  |
| Nakakubo et al (16) | Leukocytes (WBC)  Eosinophils  Procalcitonin  CT imaging score  Alternative diagnosis | Score | / | COVID-19 Clinical Risk score (0 – 11)  Cut-off 0-4 for low risk; 5-7 moderate risk and 8-11 for high risk |
|  |  |  |  |  |
| Fink et al (17) | Age  Fever  Maximal FiO2  CRP  Normal chest X-ray  Neutrophils | Binary logistic regression | Maximal FiO2  Replaced by the median value give in the original article: 0.36 | Logistic binary regression model |
|  |  |  |  |  |

| **Table A2. Sensitivity, specificity, predictive negative and positive value for scores from selected articles with cut-off values** | | | | | | | | | |
| --- | --- | --- | --- | --- | --- | --- | --- | --- | --- |
|  |  | **Wave 1 (patients from Wave 2 and between waves were discarded)** | | | | **Wave 2 (patients from wave 1 and between waves were discarded)** | | | |
|  |  | **Se (95CI%)** | **Sp (95CI%)** | **PPV (95CI%)** | **NPV (95CI%)** | **Se (95CI%)** | **Sp (95CI%** | **PPV (95CI%** | **NPV (95CI%)** |
| ***Vieceli et al.*** |  |  |  |  |  |  |  |  |  |
| Cut-off value | ≥ 5 | 0.87 (0.81 – 0.93) | 0.57 (0.53 – 0.62) | 0.40 (0.35 – 0.46) | 0.93 (0.90 – 0.96) | 0.82 (0.78 – 0.86) | 0.57 (0.54 – 0.64) | 0.59 (0.55 – 0.64) | 0.81 (0.76 – 0.85) |
| ***Tordjman et al.*** |  |  |  |  |  |  |  |  |  |
| Cut-off value | ≥ 0 | 100 |  |  |  | 100 |  |  |  |
|  | ≥ 1 | 0.97 (0.92 – 0.99) | 0.17 (0.14 – 0.21) | 0.28 (0.24 – 0.32) | 0.94 (0.86 – 0.98) | 0.97 (0.95 – 0.98) | 0.15 (0.12 – 0.19) | 0.47 (0.43 – 0.50) | 0.87 (0.77 – 0.93) |
|  | ≥ 2 | 0.94 (0.89 – 0.97) | 0.32 (0.28 – 0.37) | 0.31 (0.27 – 0.36) | 0.94 (0.89 – 0.97) | 0.91 (0.87 – 0.93) | 0.32 (0.27 – 0.36) | 0.50 (0.46 – 0.54) | 0.81 (0.75 – 0.87) |
|  | ≥ 3 | 0.84 (0.77 – 0.89) | 0.51 (0.46 – 0.56) | 0.36 (0.31 – 0.41) | 0.90 (0.86 – 0.94) | 0.80 (0.75 – 0.84) | 0.51 (0.46 – 0.55) | 0.55 (0.51 – 0.60) | 0.77 (0.72 – 0.81) |
|  | ≥ 4 | 0.69 (0.61 – 0.77) | 0.70 (0.65 – 0.74) | 0.43 (0.37 – 0.50) | 0.87 (0.83 – 0.91) | 0.63 (0.57 – 0.68) | 0.72 (0.67 – 0.76) | 0.63 (0.58 – 0.68) | 0.71 (0.67 – 0.75) |
|  | ≥ 5 | 0.25 (0.18 – 0.32) | 0.95 (0.93 – 0.97) | 0.62 (0.48 – 0.74) | 0.79 (0.76 – 0.83) | 0.30 (0.25 – 0.35) | 0.94 (0.92 – 0.96) | 0.80 (0.72 – 0.86) | 0.64 (0.60 – 0.67) |
| ***Kurstjens et al.*** |  |  |  |  |  |  |  |  |  |
| Cut-off value | > 2 | 0.96 (0.93 – 0.99) | 0.33 (0.28 – 0.37) | 0.32 (0.28 – 0.37) | 0.96 (0.93 – 0.99) | 0.95 (0.93 – 0.97) | 0.33 (0.28 – 0.37) | 0.52 (0.48 – 0.56) | 0.90 (0.85 – 0.95) |
|  | > 3 | 0.94 (0.90 – 0.98) | 0.43 (0.38 – 0.47) | 0.35 (0.30 – 0.40) | 0.96 (0.93 – 0.99) | 0.92 (0.90 – 0.95) | 0.43 (0.39 – 0.48) | 0.56 (0.52 – 0.60) | 0.88 (0.84 – 0.93) |
|  | > 4 | 0.92 (0.87 – 0.97) | 0.55 (0.50 – 0.60) | 0.40 (0.35 - 0.46) | 0.95 (0.93 – 0.98) | 0.89 (0.86 – 0.93) | 0.55 (0.50 – 0.59) | 0.60 (0.56 – 0.64) | 0.87 (0.83 – 0.91) |
|  | ≥ 5 | 0.89 (0.84 – 0.95) | 0.65 (0.61 – 0.70) | 0.46 (0.40 – 0.52) | 0.95 (0.92 – 0.98) | 0.84 (0.80 – 0.88) | 0.67 (0.63 – 0.71) | 0.66 (0.62 – 0.71) | 0.85 (0.81 – 0.89) |
|  | ≥ 9 | 0.68 (0.60 – 0.76) | 0.87 (0.84 – 0.90) | 0.64 (0.56 – 0.71) | 0.89 (0.86 – 0.92) | 0.66 (0.61 – 0.71) | 0.91 (0.88 – 0.93) | 0.84 (0.80 – 0.89) | 0.78 (0.74 – 0.81) |
|  | ≥ 10 | 0.61 (0.53 – 0.70) | 0.91 (0.88 – 0.94) | 0.69 (0.60 - 0.77) | 0.88 (0.85 – 0.91) | 0.55 (0.49 – 0.60) | 0.94 (0.91 – 0.96) | 0.87 (0.82 – 0.91) | 0.73 (0.69 – 0.77) |
|  | ≥ 11 | 0.52 (0.44 – 0.60) | 0.94 (0.91 – 0.96) | 0.73 (0.65 – 0.82) | 0.86 (0.82 – 0.89) | 0.43 (0.38 – 0.49) | 0.96 (0.94 – 0.98) | 0.89 (0.84 – 0.94) | 0.69 (0.65 – 0.72) |
|  | ≥ 12 | 0.39 (0.31 – 0.48) | 0.97 (0.95 – 0.99) | 0.83 (0.73 – 0.92) | 0.83 (0.80 – 0.86) | 0.33 (0.28 – 0.38) | 0.97 (0.96 – 0.99) | 0.90 (0.84 – 0.95) | 0.66 (0.62 – 0.69) |
| ***Aldobyany et al.*** |  |  |  |  |  |  |  |  |  |
| Cut-off value | ≥ 4 | 0.70 (0.63 – 0.78) | 0.51 (0.46 – 0.55) | 0.32 (0.27 – 0.37) | 0.84 (0.79 – 0.88) | 0.69 (0.64 – 0.74) | 0.45 (0.40 – 0.49) | 0.49 (0.44 – 0.53) | 0.65 (0.60 – 0.70) |
|  | ≥ 3 | 0.82 (0.76 – 0.89) | 0.28 (0.24 – 0.33) | 0.27 (0.23 – 0.32) | 0.83 (0.76 – 0.89) | 0.79 (0.75 – 0.84) | 0.27 (0.23 – 0.31) | 0.45 (0.42 – 0.50) | 0.64 (0.57 – 0.70) |
| ***Nakakubo et al.*** |  |  |  |  |  |  |  |  |  |
| Cut-off value | > 5 | 0.88 (0.83 – 0.94) | 0.70 (0.66 – 0.75) | 0.49 (0.43 – 0.56) | 0.95 (0.92 – 0.97) | 0.73 (0.68 – 0.78) | 0.61 (0.57 – 0.66) | 0.59 (0.55 – 0.64) | 0.75 (0.71 – 0.80) |

| **Table A3. AUROC and calibration measures for scores and formulas from selected articles with cut-off values** | | | | | | | | |
| --- | --- | --- | --- | --- | --- | --- | --- | --- |
|  | **Wave 1** | | | | **Wave 2** | | | |
|  |  |  | **Calibration** | |  |  | **Calibration** | |
|  | **AUROC (95CI%)** | **Brier score** | **Slope (95CI%)** | **Intercept (95CI%)** | **AUROC (95CI%)** | **Brier score** | **Slope (95CI%)** | **Intercept (95CI%)** |
| ***Vieceli et al.*** |  |  |  |  |  |  |  |  |
| Score (0-9) | 0.77  (0.75 – 0.80) |  |  |  | 0.76  (0.74 – 0.79) |  |  |  |
| Model | 0.77  (0.72 – 0.82) | 0.16 | 0.57  (0.45 – 0.69) | 0.00  (-0.24 – 0.24) | 0.76  (0.73 – 0.80) | 0.20 | 0.52  (0.43 – 0.60) | 0.00  (-0.18 – 0.18) |
| ***Tordjman et al.*** |  |  |  |  |  |  |  |  |
| Score (0-5) | 0.75  (0.70 – 0.79) |  |  |  | 0.73  (0.69 – 0.76) |  |  |  |
| Model | 0.75  (0.71 – 0.80) | 0.16 | 0.46  (0.35 – 0.56) | 0.00  (-0.24 – 0.24) | 0.74  (0.70 – 0.77) | 0.23 | 0.38 (0.31 – 0.45) | 0.00  (-0.20 – 0.20) |
| ***Kurstjens et al.*** |  |  |  |  |  |  |  |  |
| Score (0-14) | 0.86  (0.81 – 0.89-) |  |  |  | 0.85  (0.22 – 0.88) |  |  |  |
| ***Aldobyany et al.*** |  |  |  |  |  |  |  |  |
| Score (0-8) | 0.62  (0.56 – 0.67) |  |  |  | 0.58  (0.54 – 0.62) |  |  |  |
| ***Nakakubo et al.*** |  |  |  |  |  |  |  |  |
| Score (0-11) | 0.88  (0.83 – 0.91) |  |  |  | 0.74  (0.71 – 0.78) |  |  |  |
| ***Fink et al.*** |  |  |  |  |  |  |  |  |
| Model | 0.78  (0.73 – 0.82) | 0.15 | 1.08  (0.85 – 1.31) | 0.00  (-0.21 – 0.21) | 0.78  (0.75 – 0.81) | 0.19 | 1.10  (0.91 – 1.29) | 0.00  (-0.15 – 0.15) |

| **Table A4. Descriptive analysis for all parameters used in scores/formulas, globally and by time period** | | | | | | | |
| --- | --- | --- | --- | --- | --- | --- | --- |
|  |  | **All** | **Wave 1** | **Between waves** | **Wave 2** |  | **p-value** |
| ***Demographics*** |  |  |  |  |  |  |  |
| Age (years) | N | 1618 | 588 | 204 | 826 |  |  |
|  | P50 (P25 – P75) | 73.00 (62.00-82.00) | 72.00 (60.00 – 82.00) | 73.00 (61.00 – 82.00) | 73.00 (63.00 – 82.00) |  | 0.40* |
| Gender | N | 1618 |  |  |  |  |  |
|  | Male | 883 (54.6) | 319 (54.3) | 103 (50.5) | 461 (55.8) |  | 0.39** |
|  | Female | 735 (45.4) | 269 (45.7) | 101 (49.5) | 365 (44.2) |  |  |
| ***Comorbidities*** |  |  |  |  |  |  |  |
| Cardiac disease | N | 593 | 338 | 24 | 231 |  |  |
|  | Yes | 200 (33.7) | 130 (38.5) | 5 (20.8) | 65 (28.1) |  | 0.015** |
| Immunosuppression | N | 593 | 338 | 24 | 231 |  |  |
|  | Yes | 38 (6.4) | 28 (8.3) | 2 (8.3) | 8 (3.5) |  | 0.065** |
| Renal failure | N | 594 | 338 | 24 | 232 |  |  |
|  | Yes | 48 (8.1) | 33 (9.8) | 1 (4.2) | 14 (6.0) |  | 0.21** |
| ***Symptoms*** |  |  |  |  |  |  |  |
| Fever | N | 608 | 397 | 24 | 187 |  |  |
|  | Yes | 282 (46.4) | 158 (39.8) | 15 (62.5) | 109 (58.3) |  | <0.0001** |
| Dry cough | N | 607 | 397 | 23 | 187 |  |  |
|  | Yes | 220 (36.2) | 132 (33.2) | 12 (52.2) | 76 (40.6) |  |  |
| Wet cough | N | 607 | 397 | 23 | 187 |  | 0.060** |
|  | Yes | 106 (17.5) | 70 (17.6) | 2 (8.7) | 34 (18.2) |  |  |
| Dyspnea | N | 608 | 397 | 24 | 187 |  | 0.52** |
|  | Yes | 403 (66.3) | 258 (65.0) | 16 (66.7) | 129 (69.0) |  |  |
| Diarrhea | N | 607 | 397 | 23 | 187 |  | 0.63** |
|  | Yes | 128 (21.1) | 77 (19.4) | 6 (26.1) | 45 (24.1) |  | 0.36** |
| ***Biological parameters*** |  |  |  |  |  |  |  |
| LDH U/L | N | 1618 | 588 | 204 | 826 |  |  |
|  | P50 (P25 – P75) | 275 (219.0 – 368.0) | 267.50 (213.50 – 357.50) | 247.00 (201.00 – 314.00) | 290.00 (230.00 – 397.00) |  | <0.0001* |
| CRP mg/L | N | 1618 | 588 | 204 | 826 |  |  |
|  | P50 (P25 – P75) | 60.40 (15.10-140.9) | 58.85 (13.65 – 133.75) | 56.90 (8.40 – 140.00) | 63.15 (19.40 – 144.60) |  | 0.032* |
| Procalcitonin µg/L | N | 1615 | 588 | 202 | 825 |  |  |
|  | P50 (P25 – P75) | 0.10 (0.04-0.34) | 0.10 (0.04 – 0.35) | 0.09 (0.03 – 0.30) | 0.11 (0.05 – 0.34) |  | 0.17* |
| Lymphocytes 10³/mm³ | N | 1618 | 588 | 204 | 826 |  |  |
|  | P50 (P25 – P75) | 1.01 (0.66-1.57) | 1.07 (0.69 – 1.65) | 1.15 (0.74 – 1.85) | 0.95 (0.63 – 1.42) |  | <0.0001* |
| Basophils 10³/mm³ | N | 1618 | 588 | 204 | 826 |  |  |
|  | P50 (P25 – P75) | 0.03 (0.01-0.05) | 0.03 (0.01 – 0.05) | 0.04 (0.02 – 0.05) | 0.03 (0.01 – 0.05) |  | 0.0048* |
| Eosinophils 10³/mm³ | N | 1618 | 588 | 204 | 826 |  |  |
|  | P50 (P25 – P75) | 0.03 (0.01-0.12) | 0.04 (0.01 – 0.13) | 0.05 (0.01 – 0.16) | 0.03 (0.00 – 0.11) |  | 0.0039* |
| Ferritin µg/L | N | 1614 | 588 | 203 | 823 |  |  |
|  | P50 (P25 – P75) | 332.5 (145.1-762.7) | 300.99 (145.13 – 762.70) | 255.37 (113.04 – 510.32) | 380.76 (159.75 – 852.67) |  | <0.0001* |
| Leukocytes 10³/mm³ | N | 1618 | 588 | 204 | 826 |  |  |
|  | P50 (P25 – P75) | 9.40 (6.61-12.76) | 9.53 (7.02 – 12.51) | 10.51 (7.62 – 14.41) | 8.92 (6.03 – 12.47) |  | 0.0002* |
| Neutrophils 10³/mm³ | N | 1618 | 588 | 204 | 826 |  |  |
|  | P50 (P25 – P75) | 7.09 (4.68-10.28) | 7.35 (4.87 – 10.08) | 7.96 (5.09 – 11.48) | 6.90 (4.54 – 10.20) |  | 0.018* |
| ***Chest X-ray*** |  |  |  |  |  |  |  |
| Radiological anomaly | N | 1475 | 571 | 173 | 561 |  | 0.0018** |
|  | No | 294 (19.9) | 101 (18.7) | 52 (30.1) | 141 (18.5) |  |  |
|  | Yes | 1181 (80.1) | 440 (81.3) | 121 (69.9) | 620 (81.5) |  |  |
| Finding | N | 1475 | 571 | 173 | 561 |  |  |
|  | No atypical signs | 825 (55.9) | 290 (53.6) | 125 (72.3) | 410 (53.9) |  | 0.0002** |
|  | Subpleural or lower lung dominant distribution | 104 (7.1) | 43 (7.9) | 7 (4.0) | 54 (7.1) |  |  |
|  | Multilobar or bilateral lesion | 458 (31.1) | 169 (31.2) | 31 (17.9) | 258 (33.9) |  |  |
|  | GGO with or without consolidation | 88 (6.0) | 39 (7.2) | 10 (5.8) | 39 (5.1) |  |  |
| Alternative diagnosis | N | 1618 | 588 | 204 | 826 |  | <0.0001** |
|  | More ikely other diagnosis | 309 (19.1) | 308 (52.4) | 0 (0.0) | 1 (0.1) |  |  |
|  | Hard to determine | 1055 (65.2) | 28 (4.8) | 204 (100.0 | 823 (99.6) |  |  |
|  | More likely COVID-19 | 254 (15.7) | 252 (42.9) | 0 (0.0) | 2 (0.2) |  |  |
| Other finding | N | 1475 | 571 | 173 | 561 |  |  |
|  | No infiltrate | 861 (58.4) | 305 (56.4) | 130 (75.1) | 426 (56.0) |  | <0.0001** |
|  | Unilateral infiltrate | 129 (8.7) | 52 (9.6) | 12 (6.9) | 65 (8.5) |  |  |
|  | Bilateral infiltrate | 485 (32.9) | 184 (34.0) | 31 (17.9) | 270 (35.5) |  |  |

*Kruskal-Wallis test ** Chis-squared test
